# Effects of yam varieties on flour physicochemical characteristics and resultant instant *fufu* pasting and sensory attributes

**DOI:** 10.1038/s41598-022-22052-z

**Published:** 2022-11-23

**Authors:** Helen E. Ufondu, Busie Maziya‐Dixon, Chigozie F. Okoyeuzu, Thomas M. Okonkwo, Charles Odilichukwu R. Okpala

**Affiliations:** 1 Food and Nutrition Sciences Laboratory, International Institute of Tropical Agriculture, Oyo Road, PMB 5320, Ibadan, Oyo State Nigeria; 2 Department of Food Science and Technology, University of Nigeria Nsukka, Nsukka, Enugu State Nigeria; 3 Department of Functional Food Products Development, Wrocław University of Environmental and Life Sciences, 51-630 Wrocław, Poland; 4 UGA Cooperative Extension, College of Agricultural and Environmental Sciences, University of Georgia, Athens, GA 30602 United States

**Keywords:** Sustainability, Carbohydrates

## Abstract

Challenges and constraints deter the effective gathering of adequate information specific to the characteristics of yam (*Dioscorea rotundata*) landraces capable of producing a promising instant pounded *fufu* product. This current work, therefore, investigated the effects of yam varieties on flour physicochemical characteristics and resultant instant *fufu* pasting and sensory attributes. The *D. rotundata* varieties, obtained from the International Institute of Tropical Agriculture (IITA) (yam-breeding programme) experimental plots, included commercially available *Fekatsa* (control)*,* alongside ten (N = 10) improved (varieties) namely: TDr 08/00068, TDr 10/00912, TDr 89/02665, TDr 95/01932, TDr 95/18544, TDr 97/00632, TDr 97/00917, TDr Agwekachi, TDr Ebute, and TDr Meccakusa). Compared to control, the improved yam varieties produced promising characteristic range values, for instance, amylose (15.77–33.89%), bulk density (0.77–0.86 g/cm^3^), setback (99.5–503.46 RVU), peak time (4.93–7.00 min) along with peak temperature (83.99 °C). From the correlation coefficient and principal component analysis, it was possible to deduce how flour physicochemical characteristics were associated with the resultant instant *fufu* pasting and sensory attributes. Considering the totality of results of this current study, both TDr 10/00912, and TDr Meccakusa yam varieties demonstrate a high potential on instant pounded flour towards producing an acceptable quality and promising *fufu* product.

## Introduction

Yam has been considered among multi-species motocotyledonous plant crop widely disseminated across the globe, from Africa, to Asia, up to Oceania and South America. The yam genus *Dioscorea* comprises about 600 species, with only a few being cultivated for food and income^1^. In fact, over 90% of the global yam (*Dioscorea* spp.) population rests in Africa^2^, wherein the West Africa leads because of Nigeria, which sustains over 10 tonnes tuber/ha^3,4^. Further, yam steadily serves as the key carbohydrate as well as income resource in the West Africa sub-region^5^. Besides smallholder farmers dominating the West Africa yam value chain, promoting its production and utilization must continue to enhance food security and poverty alleviation^3^. Besides, yam given its essential dietary contents/nutrients steadily remains nutritionally superior over other tropical root crops^2^. Yams have higher dietary protein (1–2%) and are better sources of potassium. Traditional foods in Nigeria derived from yam tubers include chips, *amala*, and pounded yam/*fufu*. In particular, the pounded yam/*fufu* occupies a very prominent space with its consumption sweeping across several ethnic groups^2^. Besides the preservation of food products directly relating to technological advancements, the upgrading of traditional food processing techniques continues to impact on food/nutrition insecurity in the West Africa sub-region^5^.

Yam remains among highly esteemed food crops largely integrated into the cultural, economic, religious, and social aspects of West Africans, besides being incorporated into the diets across several communities particularly within the tropics and subtropics^1,3^. Starch together with other constituents like fiber, lipids, and moisture in yam^3^ contribute to making effective its poundability. Additionally, the instantization processing of yam appears increasingly employed to make its starch readily digestible. The *fufu *production typically involves either the more traditional pounding of the yam with a pestle mortar with the intermittent addition of water^5^ or the less traditional use of instant pounded yam flour^6^. Specifically, when the yam tuber is boiled and pounded in a mortar, its starchy nature allows the paste to bind^7^. The production of instant yam flour should be easy, as it entails slicing, parboiling, and milling of the (yam) product, which helps to enable the production of the flour. Also, the processing facility required to implement the production of the yam flour locally in Nigeria has become increasingly available^5^. The reconstitution of instant pounded yam flour in boiled water to rapidly make the *fufu* (locally referred to as poundo yam) has become very popular. The further utilization of yam food product by consumers, farmers, and processors are mainly guided by both flour and tuber physicochemical, functional and sensory aspects^8^. Additionally, other authors like Baah et al.^9^ have characterised the physicochemical and pasting properties of typical yam, as it associated with the consumer quality of pounded yam.

Major yam species in Nigeria, applicable to West Africa sub-region, include *Dioscorea alata*/*rotundata* (white yam), *Dioscorea bulbifera* (aerial yam or air potato), *Dioscorea cayensis* (yellow yam), *Dioscorea dumentorium* (trifoliate yam), as well as *Dioscorea esculenta* (Chinese yam)^2^. However, the above-mentioned *Dioscorea* species have evolved over the years into today’s commercially available varieties. Besides Nigeria remaining among the top five countries in terms of total area under yam cultivation globally^1,10^, there are empirical breeding methods and collaborative efforts of national yam breeding programmes like those found in the International Institute of Tropical Agriculture (IITA), where considerable efforts to enhance and fortify yam varieties, specifically to make them widely adaptable to farmers, have consistently persevered. And such efforts regards the improved yam varieties have involved the selection of traits of interest based on the phenotypic expression, despite the fundamental biological constraints that impede their genetic elucidation^1^. Among the principal aims of yam breeders in Nigeria has been to produce hybrids that would particularly deliver an enhanced nutritionally consistent, stable, and quality instant pounded *fufu* product. Moreover, the extent to which yam varieties would be effective on flour production particularly to actualize a mealy, appropriately elastic, and smooth dough for the *fufu* product^5,11^ still requires further investigations. Besides, key sensorial attributes that determine the acceptability of pounded *fufu* product can include mouldability (ease of molding), springiness, as well as mass texture (visco-elasticity), whereas its rejection could be due to either lumpiness and or lack cohesiveness^12,13^. Indeed, there are still challenges and constraints that deter the effective gathering of adequate information specific to the characteristics of yam (*D. rotundata*) landraces capable of producing a promising instant pounded *fufu* product. In an attempt to provide a possible solution to one or more of the above-mentioned challenges/constraints, and importantly, to supplement existing information, the current work specifically investigated the effects of yam varieties on flour physicochemical characteristics and resultant instant *fufu* pasting and sensory attributes.

## Materials and methods

### Schematic overview of the experimental program

**Figure 1:**
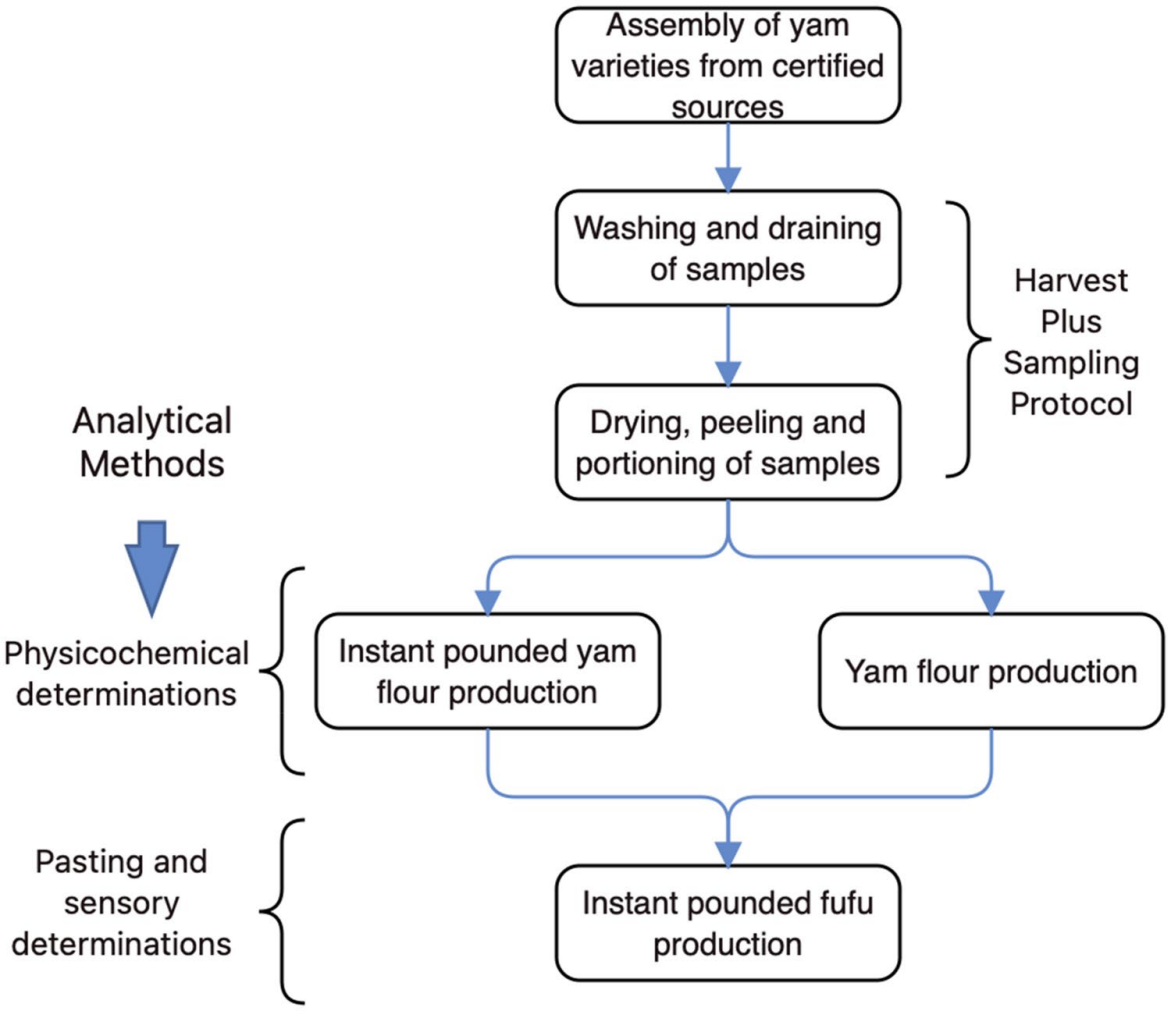
The schematic overview of the experimental program is shown in Fig. 1, from the assembly of yam varieties from certified sources, the processing stages from washing to portioning, the making of instant pounded and yam flour groups, then, instant pounded yam *fufu*, and at the same time, the analytical methods of physicochemical, as well as pasting and sensory determinations.

The schematic overview of the experimental program is shown in Fig. 1, which revealed the major stages, from the collection of yam varieties from certified sources, the processing stages from washing to portioning, the making of instant pounded and yam flour groups, then, instant pounded yam *fufu*, and at the same time. The analytical methods of physicochemical characterisation, as well as pasting and sensory determinations were carried out. For emphasis, this current work sought to further establish how varieties of yam (*D. rotundata*) play a role in the characteristics of (yam) flour, and its direct and indirect impact on the eventual pasting and sensory aspects of the emergent instant pounded *fufu* product. All the methods employed were conducted in accordance with the relevant guidelines set out by the International Institute of Tropical Agriculture (IITA), Ibadan, Nigeria. Additionally, the analytical aspects were conducted at least in duplicate measurements.

### Sample procurement, preparation and processing

*Procurement of yam varieties*: The *D. rotundata* varieties included the commercially available *Fekatsa* together with ten others, namely: TDr 08/00068, TDr 10/00912, TDr 89/02665, TDr 95/01932, TDr 95/18544, TDr 97/00632, TDr 97/00917, TDr Agwekachi, TDr Ebute and TDr Meccakusa), all locally used for pounded *fufu* production. Specifically, these above-mentioned ten (N = 10) improved *D. rotundata* varieties were procured from the IITA yam-breeding programme experimental plots (Abuja Station, Nigeria); (b) The *D. rotundata, Fekatsa ,* which served as the control, was purchased from a local market in Benue State, Nigeria.

#### Preparation and processing of yam tubers

The yam tubers, of each variety, were sorted according to their sizes, namely: big (1.0–2.0 kg), medium (0.75–0.80 kg) and small (0.5–0.6 kg) sizes. Five fresh tubers were then selected from each variety by a simple randomized procedure. The Harvest Plus sampling protocol as reported by Alamu et al.^14^ was adopted to process the yam tubers, which included washing, drain-drying, peeling and proportioning. The latter specifically involved the cutting of yam samples into four longitudinal portions from the proximal to the distal end. The entire batch per variety were divided into two portions. Whereas one served for the Instant Pounded Yam Flour (IPYF) production, the other served for the yam flour production.

### Instant pounded yam, yam flour and fufu production

#### Instant pounded yam flour production

The production of Instant Pounded Yam Flour (IPYF) followed the method described by Abiodun e*t al*.^15^ with slight modifications. The first two opposite portions of the longitudinally divided yam tubers were sliced (0.02 mm thick) and immersed in 800 ppm sodium metabisulphite (Na_2_S_2_O_5_) solution for 20 min to inhibit enzymatic browning. The sliced yams were thereafter blanched in water at 100 °C for 10 min. The sliced, sulphited and blanched yam samples were dried to 5.20% moisture content (d.b.) in a fabricated Niji Lukas cabinet dryer (Niji Lukas Nigeria Ltd., Isheri Round About Lagos Idimu, Lagos, Nigeria) at 60 °C for 72 h. The dried samples were milled into fine flour using a 0.50 mm sieve size, helped by Perten LabMill 3100 (Calibre Control International Ltd., Warrington -UK), and thereafter, transferred into polythene whirl-pack, and kept at 20 °C until required for further use.

#### Yam flour production

The yam flour production was performed to follow as much possible the method widely used across the various artisanal cottages in Nigeria. This generally involves the yam samples being longitudinally divided, and subsequently cut into cubes (approximately 1 cm thickness), then thoroughly mixed and subjected to drying. Specifically for this study, the drying was performed using AtmoSAFE Memmert air convection oven models 30–750 (Memmert GmbH + Co.KG, D-91126 Schwabach, Germany) that operated at 70 °C for 72 h. The emergent dry chips were then be milled into fine flour using a 0.50 mm sieve size, helped by Perten LabMill 3100. Subsequently, the yam flour was transferred into polythene whirl-pack, and kept at − 20 °C until required for further use.

#### Instant pounded yam fufu production

The instant pounded yam *fufu* was prepared using the method described by Babajide et al*.*^16^ with slight modification. Weights of yam flour samples of about 100 g were stirred in 500 mL of boiling water on a cooker (Polystar Standing Gas Cooker, PVFS-80EG1) continuously for about 6 min, which continued until a homogenous smooth dough was formed. Thereafter, the smooth dough was wrapped in an aluminum foil and stored in Styrofoam insulated box to keep it warm until required.

## Physicochemical characterisation of yam flour

### Amylose content determination

The amylose content of yam flour was determined as described by Udachan et al*.*^17^ with slight modifications. About 0.1 g of the yam flour samples were weighed into a test tube. To this, 1 N NaOH (9 mL) and 1 mL of 95% ethanol were carefully added and vortex with the test tube mouth covered. The mixture was subjected to heating for 10 min in a boiling water bath to gelatinize the starch and cooled to room. A 10-times dilution of the extract was made by taking 1 mL and making this up to 10 mL with 9 mL of water. From the diluents, an aliquot of 0.5 mL was taken for analysis. To this, 0.1 mL of acetic acid solution and 0.2 mL of iodine solution were added. The volume was then made up to 10 mL with 9.2 mL of distilled water. The mixture was allowed to stand for 20 min for colour development, then vortexed and absorbance was read at 620 nm in a spectrophotometer (Spectrumlab 22pc) using a quartz cuvette, and % amylose content was calculated using following formula:

$$ Amylose\;content \, = \frac{\% Absorbance\;of\;standard \times Absorbance\;of\;sample}{{Absorbance\;of\;standard}} $$

### Amylopectin and bulk density determination

The amylopectin content of yam flour was calculated by difference following the method of Nik Shanita et al.^18^ using following formula: amylopectin (%) = 100% – amylose (%). The bulk density of yam flour was determined using the method described by Oladele and Aina^19^ and expressed as g/cm^3^.

### Swelling power and solubility index determinations

The swelling power and solubility index of yam flour employed the method described by Yu et al.^20^ with slight modifications. The yam flour (0.5 g) was placed in the centrifuge tube containing 30 mL of distilled water, and then vortex mixed for about 3 min. The tube was placed in a 60 °C water bath, stirred for 30 min, cooled, and then centrifuged (Thelco GLC- 1, 60647: Chicago, USA) at 2147.0 g for 20 min. The supernatant from the centrifuge tube was transferred into an aluminum dish, and baked at 105 °C using Fisher Scientific Oven Model 655 F. There would be no precipitation after drying, which enabled the swelling power, and solubility index calculations as shown below:

$$ Swelling\;power\;\left( {g/g} \right) \, = \frac{Weight\;of\;sediment}{{Sample\;weight - Weight\;of\;soluble\;matter}} $$

$$ \% \, Solubility \, index = \frac{Weight\;of\;soluble\;matter \times 100}{{Weight\;of\;sample}} $$

### Water absorption capacity

Water absorption capacity (WAC) of yam flour was determined following the method described by Lawal and Adebowale^21^ with slight modifications. Briefly, 0.5 g of yam flour was placed into a 10 mL centrifuge tube, and 5 mL distilled water added, then mixed, and left to stand for 30 min, before centrifugation (30 min at 1207.47 g). The supernatant was decanted and the sample was kept to drain at an angle of 45° for 10 min followed by the draining of excess water in the upper phase. The tube containing the residue was then reweighed to determine the WAC, determined using the formula below:

$$ \% \, WAC \, = \frac{{final\;weight - \left( {tube\;weight + sample\;weight} \right) \times 100}}{Sample\;weight} $$

### Pasting and sensory attributes of instant pounded fufu product

#### Pasting properties

The pasting attributes of instant pounded *fufu* samples were determined using the method described by Yu et al.^20^ with slight modifications. The pasting parameters were collected with the help of the Rapid Visco Analyser (RVA), (Tecmaster TCW3, Perten Instrument, Newport Scientific Pty. Ltd, Australia). Instant pounded yam samples (~ 3.35 g sample) was mixed in 25 mL of distilled water in a previously dried canister, and thereafter the thoroughly mixed and fitted into the RVA. The samples were analysed using a 12-min standard profile, where the slurry was heated from 50 to 95 °C with a holding time of 2 min, followed by cooling to 50 °C, with another 2 min holding time. Both the heating and cooling was at a constant rate of 11.25 °C/min with constant shear at 369.70 g. The viscosity was determined as expressed in terms of Rapid Visco Units (RVU). The viscogram profile/pasting curves associate with time, viscosity and temperature. From the pasting profile connected to the RVA instrument, the pasting attributes that were determined included: (a) peak viscosity, (b) peak time (pasting time), (c) breakdown, (d) trough viscosity, (e) set back, (f) final viscosity and as well as (g) pasting temperature.

#### Sensory evaluation of fufu samples

The sensory evaluation of the instant *fufu* samples was carried out using the method described by Meilgaard et al.^22^ with slight modifications. The sensory panelists comprised ten (10) staff and graduate students of IITA—Ibadan. All panelists, already confirmed as consumers of pounded *fufu*, undertook the sensory training of evaluation criteria set out to specifically discriminate between the levels of color, smoothness, adhesiveness, hardness, springiness, and cohesiveness. The verbal consent was taken prior to the sensory evaluation, and panelists’ participation was voluntary. To ensure their privacy, no names/gender was reported. The sensory evaluation took place in well ventilated sensory booths of neutral color, proper lighting, and distraction-free. Each panelist simultaneously evaluated the samples which were labelled with Three-digit codes derived from a standard random table, using a 7-point scale, where: 1 = very much worse, 2 = worse, 3 = slightly worse, 4 = no difference, 5 = slightly better, 6 = better, 7 = very much better. In line with Çakmakçı et al.^23^, each panelist used warm water to cleanse taste palates between samples, to ensure the evaluation of previous *fufu* sample did not influence the new one.

### Statistical analysis

The data were analysed using one-way analysis of variance (ANOVA). Mean value ± standard deviation (SD) were calculated by Student–Newman–Keuls (SNK) Test. Whereas the use of Pearson’s correlation coefficient helped to deduce the potential relationships that could exist between the yam flour characteristics, and either pasting and or sensory aspects of instant pounded *fufu* product, Principal Component Analysis helped to deduce the potentially recommendable yam variety, pattern as well as emergent relationships that could exist between the various tested parameters (chemical, physicochemical, pasting and sensory) at this study. The probability level was set at *p* < 0.05 (95% confidence level). Statistical Analysis Systems (SAS) package (version 9.4. SAS Institute Inc., Cary, North Carolina—US) was used to run the data.

## Results and discussion

### Effects of yam varieties on flour physicochemical characteristics

The effects of yam varieties on flour physicochemical characteristics, obtained from control and 10 improved *D. rotundata* varieties, are presented in Table 1. The amylose, amylopectin contents, and swelling power differed significantly (*p* < 0.05) comparing control and improved *D. rotundata* varieties, with range between 15.77–33.89%, 66.11–84.23%, and 5.44–8.62% respectively. For instance, the amylose content was lowest in Meccakusa(15.77%) and highest in TDr 89/02665 (33.89%). More so, the swelling power was least at TDr 97/00632 (5.44%) and highest at Meccakusa (8.62%). Most of the improved varieties of *D. rotundata* had swelling power that was lower (5.44–7.53%) than that of *Fekatsa* (7.59%) except for Meccakusa (8.62%). Almost all improved *D. rotundata* varieties with the exception of Meccakusa (8.62%) had swelling power (5.44 to 7.53%) lower than that of *Fekatsa* (7.59%). Also, with the exception of TDr10/00912 and TDr Meccakusa varieties, the *D. rotundata* improved varieties gave amylose contents (20.90–33.89%) above the control (19.89%), and amylopectin contents (66.11–79.11%) below the control (80.11%), which appears in agreement with previously reported data^14^. Amylose content being lower than the amylopectin in *D. rotundata* varieties herein, resembled another variety reported by Okorie et al.^24^. Amylose and amylopectin differences may depict either enzymatic activity of starch biosynthesis^25^, or differences in starch during gelation^26^.

**Table 1:** Yam varietal effects on flour physicochemical characteristics as obtained from *Dioscorea rotundata* control and improved 10 varieties.

Serial no.	Variety	Amylose (%)	Amylopectin (%)	Swelling power	SolubilityI (%)	WAC* (%)	Bulk density (g/cm^3^)
1	TDr 08/00068	27.98^b^ ± 0.25	72.03f. ± 0.25	6.97^ba^ ± 0.37	6.74^de^ ± 0.12	196.95^ba^ ± 3.64	0.86^a^ ± 0.02
2	TDr10/00912	17.07f. ± 0.49	82.94^b^ ± 0.49	7.53^ba^ ± 0.16	9.64^a^ ± 0.23	223.19^a^ ± 7.56	0.83^a^ ± 0.03
3	TDr 89/02665	33.89^a^ ± 0.06	66.11^g^ ± 0.06	5.71^b^ ± 0.67	7.05^dc^ ± 0.35	170.48^dc^ ± 14.62	0.77^a^ ± 0.00
4	TDr 95/01932	20.90^e^ ± 0.45	79.11^c^ ± 0.44	5.87^b^ ± 0.16	6.78^de^ ± 0.1`3	198.34^ba^ ± 0.86	0.77^a^ ± 0.00
5	TDr 95/18544	27.12^cb^ ± 0.29	72.89^fe^ ± 0.29	6.19^b^ ± 0.56	6.23^e^ ± 0.23	165.74^cd^ ± 4.86	0.77^a^ ± 0.00
6	TDr 97/00632	33.65^a^ ± 0.29	66.36^g^ ± 0.29	5.44^b^ ± 0.05	5.46^f^ ± 0.20	163.01^cd^ ± 3.15	0.81^a^ ± 0.05
7	TDr 97/00917	26.12^c^ ± 1.32	73.88^e^ ± 1.32	6.92^ba^ ± 0.04	7.46^c^ ± 0.39	218.40^a^ ± 7.85	0.81^a^ ± 0.02
8	TDr Agwekachi	24.33^d^ ± 0.73	75.68^d^ ± 0.73	6.20^b^ ± 0.17	5.31^f^ ± 0.03	184.18^bc^ ± 15.85	0.84^a^ ± 0.02
9	TDr Ebute	24.64^d^ ± 0.00	75.36^d^ ± 0.00	6.03^b^ ± 0.16	7.53^c^ ± 0.11	144.07^d^ ± 0.04	0.86^a^ ± 0.08
10	TDr Meccakusa	15.77^g^ ± 0.52	84.23^a^ ± 0.52	8.62^a^ ± 1.88	10.14^a^ ± 0.18	226.82^a^ ± 11.47	0.84^a^ ± 0.04
	Mean	24.67	75.33	6.63	7.36	191.84	0.81
	SD	5.86	5.86	1.07	1.54	28.41	0.04
	Min value	15.77	66.11	5.44	5.31	144.07	0.77
	Max value	33.89	84.23	8.62	10.14	226.82	0.86
	CV (%)	23.75	7.78	16.14	20.92	14.81	4.93
	*Fekatsa*	19.89^e^ ± 0.00	80.11^c^ ± 0.00	7.59^ab^ ± 0.47	8.65^b^ ± 0.27	219.05^a^ ± 11.25	0.77^a^ ± 0.00

*WAC Water absorption capacity; Solubility I: Solubility Index.

^a^Parameter mean value ± SD; Means with the same superscripts in the same column are not significantly different (*p* > 0.05).

Amylopectin contains less amorphous and more crystalline regions which confers a firm network to starch molecules resulting in a firm gel with higher stability after cooking. The higher amylose and lower amylopectin observed with *D. rotundata* varieties might be as a result of the planting conditions. The amylose and amylopectin contents may also be affected by crop age or climate^27^. Starch physical properties could be influenced by planting (growing) conditions which could associate with soil temperature rise during the cultivation/growth of yam crop/tuber. Such soil temperature rise might vary the average size and gelatinization of the starch granule^26,28^. The relatively low swelling power observed with most of the improved varieties of *D. rotundata* could be due to high amylose content which might have influenced their swelling. High amylose content might be linked to low swelling power, due to high reinforcement of internal network by amylose molecules^29^. Additionally, swelling property of starch granules might be as a result of the property of amylopectin, with amylose acting as a dilutant^30^. Increased soluble amylose content of some improved varieties might facilitate interactions between amylose-amylose, amylose-amylopectin, as well as amylose–lipid, resulting in decrease of swelling power^31^. The amylose/amylopectin ratio of starches may be characterized by their functionality, and how the emergent texture of the product is affected^32^.

Table 1 also shows solubility, water absorption capacity (WAC), and bulk density (BD) of the improved yam varieties ranged between 5.31–10.14%, 144.07–226.82%, and 0.77–0.86 g/cm^3^, respectively. Additionally, significant differences (*p* < 0.05) were observed in solubility and WAC comparing control and improved *D. rotundata* varieties. However, the BD resembled (*p* > 0.05) across the improved *D. rotundata* varieties. Despite this, the BD of improved varieties of *D. rotundata* appeared considerably high relative to control. This suggests that the flour of improved *D. rotundata* was denser than *Fekatsa* flour^33^. Moreover, the bulk density would be affected by particle size. Hence, the relatively high BD of improved varieties of *D. rotundata* could be attributed to the growing conditions associated with temperature rise during growth of the tubers^26,28^. Besides, the starch physical properties could be influenced by planting (growing) conditions, which then associates with the soil temperature rise during the cultivation as well as growth of yam crop/tuber. Such soil temperature rise might contribute to vary the average size and gelatinization of the starch granule^26,28^. Bulk density values of 0.50–0.62 g/mL has been reported for the raw flour samples of *D. cayenensis, D. bulbifera and D. rotundata*^34^. Lower bulk density products portray less weight when packaged in a given volume of container^35^. Moreover, the solubility was lowest at Agwekachi (5.31%) but highest at Meccakusa (10.14%). Also, the WAC was lowest at TDr Ebute (144.07%) but highest at Meccakusa(226.82%). Most of the improved varieties of *D. rotundata* gave solubility values (5.31 to 7.53%) lower than that of *Fekatsa* (8.65%). Lower solubility of improved varieties of *D. rotundata* might be due to the protein-amylose complex formation. High solubility in starches associates with the high content of amylose, loosely linked with the macromolecular structure, to either leach or release it during the swelling process^14,36,37^. The differences in WAC between the control and improved *D. rotundata* varieties might be attributed to the differences in the amylose and amylopectin in the native granules of starch.Higher WAC observed in some improved varieties compared to *Fekatsa* may be due to loose association of starch polymers, amylose/amylopectin which, tends to absorb more water^38^ with the possible loss of starch crystalline structure^31^. The higher the WAC the greater the amount of water required to make IPYF with desirable consistency and thereby stabilize the starches against degradative effects such as syneresis^39^, which functionally assure the product cohesiveness^40,41^.

### Effects of yam varieties on pasting and sensory attributes of instant pounded fufu product

The yam varietal effects on the pasting attributes (pasting temperature, peak viscosity, pasting time, trough viscosity, breakdown viscosity, final viscosity and setback viscosity) of the instant pounded *fufu* samples obtained from control and 10 improved *D. rotundata* varieties are presented in Tables 2 and 3. The pasting temperature, peak viscosity, peak (pasting) time, trough viscosity, breakdown viscosity, final viscosity and recorded setback of the improved varieties of *D. rotundata* for consistency ranged from 81.65 to 86.43 °C, from 299.67 to 483.00 RVU, from 4.93 to 7.00 min, from 209.92 to 397.00 RVU, from 9.08 to 263.09 RVU, from 365.88 to 729.67 RVU, and from 99.50 to 503.46 RVU, respectively. Peak pasting temperature appears to be when the viscosity of the stirred instant pounded yam flour paste begins to rise/swell^42^. *Fekatsa* showed a lower pasting temperature of 81.57 °C as compared to *D. rotundata* improved varieties, resembling previously reported data^36^. Increased soluble amylose content of some improved varieties might facilitate interactions between amylose-amylose, amylose-amylopectin, as well as amylose–lipid, resulting in decrease of swelling power and pasting temperature. Therefore, *D. rotundata* varieties with higher pasting temperature may have smaller granule size and higher amylose content^43^. *Fekatsa* gave higher peak viscosity (578.21 RVU) compared to most *D. rotundata* improved varieties. Meccakusa gave peak viscosity (483.00 RVU) that compared well with the control. High peak viscosity observed with Meccakusa might be attributed to the high WAC which enabled the flour to absorb more water and swell freely during cooking. Peak viscosity is an indication of water absorption capacityand also measures the ability of starch to swell freely^6^.

**Table 2:** Yam varietal effects on the pasting attributes (peak viscosity, trough viscosity, and breakdown viscosity) of the instant pounded *fufu* samples as obtained from control(*Fekatsa*) and 10 improved varieties of *Dioscorea rotundata.*

Serial no.	Variety	Peak visc (RVU)	Trough visc (RVU)	B.Down visc (RVU)
1	TDr08/00068	426.71^ac^ ± 32.82	250.00^b^ ± 15.41	115.46^bc^ ± 80.91
2	TDr10/00912	473.00^a^ ± 6.36	300.83^ab^ ± 3.89	263.09^a^ ± 0.47
3	TDr 89/02665	398.63^c^ ± 15.97	228.17^b^ ± 19.21	138.67^bc^ ± 37.95
4	TDr 95/01932	363.55^c^ ± 28.81	235.88^b^ ± 24.46	127.67^bc^ ± 53.27
5	TDr 95/18544	327.08^cd^ ± 13.02	224.63^b^ ± 32.46	43.00^c^ ± 51.62
6	TDr 97/00632	250.17^d^ ± 14.85	241.08^b^ ± 14.85	9.08^c^ ± 0.00
7	TDr 97/00917	317.34^cd^ ± 2.60	297.29^ab^ ± 3.95	20.04^c^ ± 1.36
8	TDr Agwekachi	299.67^bc^ ± 11.79	209.92^b^ ± 5.89	18.30^c^ ± 19.62
9	TDr Ebute	353.54^cd^ ± 10.31	241.29^b^ ± 14.85	112.25^bc^ ± 47.38
10	TDr Meccakusa	483.00^a^ ± 1.41	397.00^a^ ± 28.68	214.09^ba^ ± 33.82
	Means	387.07	276.09	113.37
	SD	90.2	74.82	87.74
	Min	299.67	209.92	9.08
	Max	473	397	263.09
	CV (%)	23.3	27.1	77.39
	*Fekatsa*	578.21^a^ ± 100.94	435.33^a^ ± 7.72	185.42^a^ ± 6.60

Parameter mean value ± SD; Values with the same superscripts in the same column are not significantly different (*p* > 0.05); *Visc* Viscosity, *B. Down* breakdown, *RVU* Rapid Visco Analyser Unit.

**Table 3:** Yam varietal effects on other pasting attributes (pasting temperature, final viscosity and setback viscosity) of the instant pounded *fufu* samples as obtained from control(*Fekatsa*) and 10 improved varieties of *Dioscorea rotundata.*

Serial no.	Variety	Final visc (RVU)	Setback(RVU)	Peak time (Min)	Pasting temp (^o^C)
1	TDr08/00068	673.13^ba^ ± 5.83	423^bac^ ± 32.07	5.14^b^ ± 0.09	83.30^cb^ ± 0.00
2	TDr10/00912	713.38^ba^ ± 45.32	124.79^e^ ± 6.42	5.04^b^ ± 0.05	81.65^d^ ± 0.00
3	TDr 89/02665	631.65^b^ ± 3.15	482.04^ab^ ± 113.43	5.43^ba^ ± 0.14	83.30^cb^ ± 0.00
4	TDr 95/01932	491.50^c^ ± 32.06	255.63^be^ ± 56.50	5.20^b^ ± 0.18	84.10^b^ ± 0.07
5	TDr 95/18544	635.67^b^ ± 27.58	312.00^ae^ ± 135.17	6.10^ba^ ± 1.09	85.65^a^ ± 0.07
6	TDr 97/00632	365.88^d^ ± 21.28	503.46^a^ ± 39.42	6.97^a^ ± 0.05	86.43^a^ ± 0.04
7	TDr 97/00917	396.79^d^ ± 6.77	378.67^ad^ ± 69.42	7.00^a^ ± 0.00	85.63^a^ ± 0.04
8	TDr Agwekachi	460.59^c^ ± 39.12	179.21^ed^ ± 70.53	6.17^ba^ ± 1.18	85.63^a^ ± 0.04
9	TDr Ebute	499.13^c^ ± 9.61	257.84^be^ ± 67.30	5.07^b^ ± 0.28	84.10^b^ ± 0.07
10	TDr Meccakusa	729.67^a^ ± 42.07	99.50^e^ ± 2.83	4.93^b^ ± 0.04	82.50^d^ ± 1.13
	Means	569.76	293.66	5.63	83.99
	SD	128.76	144.05	0.86	1.67
	Min	365.88	99.5	4.93	81.65
	Max	729.67	503.46	7	86.43
	CV (%)	22.6	49.05	15.2	1.99
	*Fekatsa*	670.00^ba^ ± 33.83	131.17^b^ ± 4.77	4.90^b^ ± 0.04	81.57^d^ ± 0.04

Parameter mean value ± SD; Values with the same superscripts in the same column are not significantly different (*p* > 0.05); Visc = Viscosity; temp = temperature; RVU = Rapid Visco Analyser Unit.

Pasting time is among the important indicators that facilitate the establishment of a good quality instant pounded yam flour^44^. In this current work, the control *Fekatsa* variety produced a comparatively lower pasting time but higher trough viscosity than the improved *D. rotundata* varieties*,* somewhat resembling data reported for other yam varieties TDa 98/01168 and TDa 99/00199(66.85 to 258.65 RVU), which appears to agree with data previously reported elsewhere^45^. High trough viscosity (holding strength) herein may be attributed to higher amylopectin content observed with the yam varieties, portraying increased water binding sites that form stable gels. Improved *D. rotundata* varieties produced a comparatively high breakdown viscosity. High breakdown viscosity at some *D. rotundata* varieties herein might associate with the high WAC which enable the starch to swell freely before their physical breakdown^6^. High final viscosity observed in some improved *D. rotundata* varieties might be due to higher amylose and amylopectin^38^, resembling those reported about achi starch when compared with its flour^46^. Setback viscosity indicates the ability of the gelatinized starch to undergo retrogradation/syneresis after cooling to 50 °C. The difference in setback viscosity values in some improved *D. rotundata* varieties might signal the starch retrogradation, which would associate more with amylose given its short length branch^9,47^.

The effects of yam varieties on the sensory attributes of instant pounded *fufu* product obtained from control and 10 improved *D. rotundata* varieties can be seen in Table 4. The results showed that variety significantly (*p* < 0.05) affected the sensory attributes of instant pounded yam *fufu*. Generally, the sensory attributes showed significant differences (*p* < 0.05) more at adhesiveness, color, cohesiveness (moldability), hardness, springiness, and less at smoothness. Whereas TDr 08/00068 (score = 3.2), TDr 89/02665 (score = 4.2), TDr 97/00632 (score = 3.3) and TDr Ebute (score = 4.0) resembled (*p* > 0.05) the control/reference *Fekatsa* (score = 4.0), the TDr Meccakusa (score = 6.30) and TDr 10/00912 (score = 5.3) obtained improved color values. The order of smoothness desirability is as follows: TDr Meccakusa > TDr 10/00912 > TDr 97/00632 > TDr 89/00917 > TDr Agwekachi > TDr Ebute > TDr 95/01932 > TDr 95/18544 > TDr 08/00068. The TDr Meccakusa, TDr 10/00912, TDr 89/02665 and TDr 97/00632 exhibited a more desirable adhesiveness scores compared to the other improved yam varieties, and control. The cohesiveness of instant pounded yam *fufu* from all the improved yam varieties seemed less (scores = 1.8–3.9) compared to control *Fekatsa* (scor = 4.0), except TDr Meccakusa (score = 5.80), followed by TDr 10/00912 (score = 4.0). The order of cohesiveness is as follows: TDr Meccakusa > TDr 10/00912 > TDr 08/00068 > TDr 97/00632 > TDr 95/01932 > TDr 97/00917 > TDr 95/18544 > TDr 89/02665 > TDr Ebute > TDr Agwekachi. The springiness of TDr Meccakusa (score = 5.5) significantly higher (*p* < 0.05) compared to other improved yam varieties, and the control. However, the springiness of TDr 08/00068 (score = 4.1), TDr 10/00912 (score = 4.1) and TDr 97/00632 (score = 4.1) resembled (*p* > 0.05) the control *Fekatsa* (score = 4.0). The hardness of instant pounded yam *fufu* of TDr Meccakusa (score = 5.7) followed by TDr 10/00,912 (score = 5.30), were both significantly higher (*p* < 0.05) compared with those of other improved varieties, and the control (score = 4.0).

**Table 4:** Yam varietal effects on the sensory attributes of instant pounded *fufu* product as obtained from control and 10 improved varieties of *Dioscorea rotundata.*

Serial no.	Variety	Color	Smoothness	Adhesiveness	Cohesiveness	Springiness	Hardness
1	TDr 08/00068	3.20^bcde^ ± 2.07	4.00^b^ ± 1.59	4.30^bc^ ± 1.77	3.90^c^ ± 1.48	4.10^b^ ± 1.03	4.90^abc^ ± 1.64
2	TDr10/00912	5.30^a^ ± 1.97	4.70^b^ ± 1.59	4.60^b^ ± 2.05	4.00^b^ ± 2.05	4.10^b^ ± 1.03	5.30^ab^ ± 1.05
3	TDr 89/02665	4.20^b^ ± 1.41	3.90^b^ ± 0.82	4.60^b^ ± 1.75	1.90^d^ ± 1.07	1.90^c^ ± 1.56	4.20^bcd^ ± 1.62
4	TDr 95/01932	2.60^cde^ ± 1.08	4.20^b^ ± 1.81	3.90^bc^ ± 1.29	2.40^d^ ± 2.12	1.80^c^ ± 1.27	3.70^cd^ ± 1.58
5	TDr 95/18544	2.00^e^ ± 1.52	4.10^b^ ± 1.71	3.80^bc^ ± 1.69	2.00^d^ ± 1.99	1.60^c^ ± 1.18	4.40^abcd^ ± 1.07
6	TDr 97/00632	3.30^bcd^ ± 1.76	4.60^b^ ± 1.26	4.50^b^ ± 1.77	3.70^c^ ± 0.84	4.10^b^ ± 1.03	3.00^d^ ± 1.42
7	TDr 97/00917	2.50^cde^ ± 1.08	4.50^b^ ± 1.40	4.60^b^ ± 1.75	2.20^d^ ± 1.78	2.00^c^ ± 0.92	3.20^d^ ± 1.25
8	TDrAgwekachi	2.10^de^ ± 0.99	4.40^b^ ± 1.93	3.40^bc^ ± 1.70	1.80^d^ ± 1.60	1.90^c^ ± 1.41	3.80^bcd^ ± 2.02
9	TDr Ebute	4.40^ba^ ± 2.07	4.30^b^ ± 1.18	3.10^c^ ± 0.71	1.90^d^ ± 1.97	1.90^c^ ± 1.55	3.70^bc^ ± 1.49
10	TDr Meccakusa	6.30^a^ ± 1.98	6.50^a^ ± 1.41	6.00^a^ ± 1.14	5.80^a^ ± 0.84	5.50^a^ ± 1.03	5.70^a^ ± 1.05
	Mean	3.54	4.47	4.25	3.12	2.99	4.17
	SD	1.73	1.42	1.46	1.62	1.51	1.65
	Min	2	4	3.1	1.8	1.6	3
	Max	6.3	6.5	6	5.8	5.5	5.7
	CV (%)	48.87	31.77	34.35	51.92	50.5	39.56
	*Fekatsa*	4.00^b^ ± 2.07	4.00^b^ ± 1.93	4.00^bc^ ± 1.70	4.00^b^ ± 2.05	4.00^b^ ± 0.92	4.00^bcd^ ± 1.25

Values are means of 10 observations; Scale of 1 to 7, where 1 = very much worse, 2 = worse, 3 = slightly worse, 4 = no difference, 5 = slightly better, 6 = better, 7 = very much better compared to instant pounded yam from *Fekatsa* (*D. rotundata*)

^a^Parameter mean value ± SD; Values with the same superscripts in the same column are not significantly different (*p* > 0.05).

### Correlation coefficient, and principal component analysis outcomes

To study how two continuous variables possibly associate, the use of correlation coefficient has been a widely embraced analytical approach. Specifically considered as a parametric test, correlation coefficient (*r*) measures the degree of (linear) association between two data sets, where its value lies between + 1 and − 1, when the absolute value is closer to 1, the stronger the correlation would be^48^. Correlation coefficients between the physicochemical, pasting, and sensory properties are shown in Table 5. Each pasting property were significantly correlated (*p* < 0.05), except the setback viscosity (*p* > 0.05). However, the pasting time and temperature were positively correlated (*p* < 0.05; r = 0.871). More so, the improved yam varieties with lower pasting temperature and time might save cooking energy and time, especially yam flour reconstitution into instant pounded yam. Additionally, the improved yam varieties with higher gelatinization temperature and time might probably possess starch granules with restricted swelling (with more amylose). Besides, the setback viscosity were negatively correlated (*p* < 0.05) with other pasting properties. High peak, trough, breakdown and final viscosities might either inhibit or lower the tendencies for the retrogradation of starch granules after the gelatinization is to be achieved. Further, whilst the swelling power and solubility index were positively correlated (*p* < 0.05) with each sensory property, the water absorption capacity and cohesiveness were positively correlated (*p* < 0.05).

**Table 5:** Correlation coefficients between physicochemical, pasting and sensory properties.

	Color	SM	Ad	CO	sg	HD	PV	TV	Bn	FV	SB	PTM	PTP	BD	SP	SI	WA	Am	AmP
Color	1																		
sm	.668*	1																	
Ad	.720*	.734*	1																
Co	.812**	.669*	.722*	1															
sg	.759**	.624*	.686*	.967**	1														
Hd	.697*	.501	.538	.668*	.562	1													
PV	.724*	.220	.411	.749**	.727*	.533	1												
TV	.545	.450	.479	.612*	.615*	.281	.777**	1											
Bn	.830**	.330	.404	.669*	.538	.751**	.804**	.468	1										
FV	.638*	.225	.400	.568	.476	.909**	.710*	.401	.802**	1									
SB	− 0.324	− 0.399	− 0.008	− 0.351	− 0.196	− 0.421	− 0.394	− 0.428	− 0.569	− 0.373	1								
PTM	− 0.565	− 0.112	− 0.057	− 0.399	− 0.316	− 0.669*	− 0.672*	− 0.344	− 0.850**	− 0.769**	0.565	1							
PTP	− 0.745**	− 0.174	− 0.313	− 0.607*	− 0.518	− 0.687*	− 0.881**	− 0.563	− 0.955**	− 0.816**	0.513	0.871**	1						
BD	0.090	0.117	0.015	0.274	0.362	0.330	0.005	− 0.119	0.036	0.144	0.146	− 0.184	− 0.091	1					
SP	0.618*	0.624*	0.615*	0.722*	0.645*	0.643*	0.612*	0.762**	0.576	0.571	− 0.515	− 0.357	− 0.598	0.227	1				
SI	0.849**	0.576	0.575	0.706*	0.576	00.671*	0.729*	0.682*	0.869**	0.665*	− 0.570	− 0.615*	− 0.815**	− 0.007	0.819**	1			
WA	0.472	0.434	0.593	0.647*	0.538	0.467	0.572	0.640*	0.535	0.415	− 0.521	− 0.246	− 0.558	0.025	0.846**	0.668*	1		
AM	− 0.481	− 0.534	− 0.221	− 0.549	− 0.387	− 0.554	− 0.497	− 0.541	− 0.669*	− 0.468	0.946**	0.571	0.605*	− 0.009	− 0.730*	− 0.747**	− 0.685*	1	
AMP	0.506	0.528	0.245	0.570	0.403	0.560	0.528	0.561	0.698*	0.487	− 0.928**	− 0.588	− 0.631*	− 0.005	0.738**	0.781**	0.690*	− 0.996**	1

*Sm* smoothness, *Ad* adhesiveness, *Co* cohesiveness, *sg* springiness, *Hd* hardness, *PV* peak viscosity, *TV* trough viscosity, *Bn* Breakdown viscosity, *FV* final viscosity, *SB* setback viscosity, *PTM* peak time, *PTP* peak temperature, *Sp* swelling power, *SI* solubility index, *WAC* water absorption capacity, *BD* Bulk density, *AM* amylose, *AMP* amylopectin.

**Correlation is significant at the 0.01 level; *Correlation is significant at the 0.05 level.

On one hand, the amylose showed negative significant correlation (*p* < 0.05) with swelling power (r = − 0.730), solubility index (r = − 0.747), and water absorption capacity (r = − 0.685). On the other hand, the amylopectin showed positive significant correlation (*p* < 0.05) with swelling power (r = 0.738), solubility index (r = 0.781) and water absorption (r = 0.690). The lower amylose might associate directly with a higher amylopectin in the yam starch, but however, indirectly through the swelling power, solubility index, and water absorption capacity, to favor the more desirable eating qualities. Interestingly, color attribute showed positive correlation (*p* < 0.05) with breakdown (r = 0.830), final (r = 0.638), as well as peak (r = 0.724)viscosities. Despite this, other statistically significant correlations (*p* < 0.05) were found, for instance, whilst as pasting time negatively correlated with hardness (r = − 0.669), the pasting temperature negatively correlated with color (r = − 0.745), cohesiveness (r = − 0.607) and hardness (r = − 0.687). However, the setback viscosity (which measures relative instability of starch on cooking), pasting time (a measure of minimum time for gelatinization of starch) and pasting temperature (a measure of minimum temperature for gelatinization of starch) appeared not significantly correlated (*p* > 0.05) with the sensory parameters. Further, the sensory texture properties (smoothness, adhesiveness, cohesiveness, springiness and hardness) were positively correlated (*p* < 0.05) with one another.

**Figure 2:**
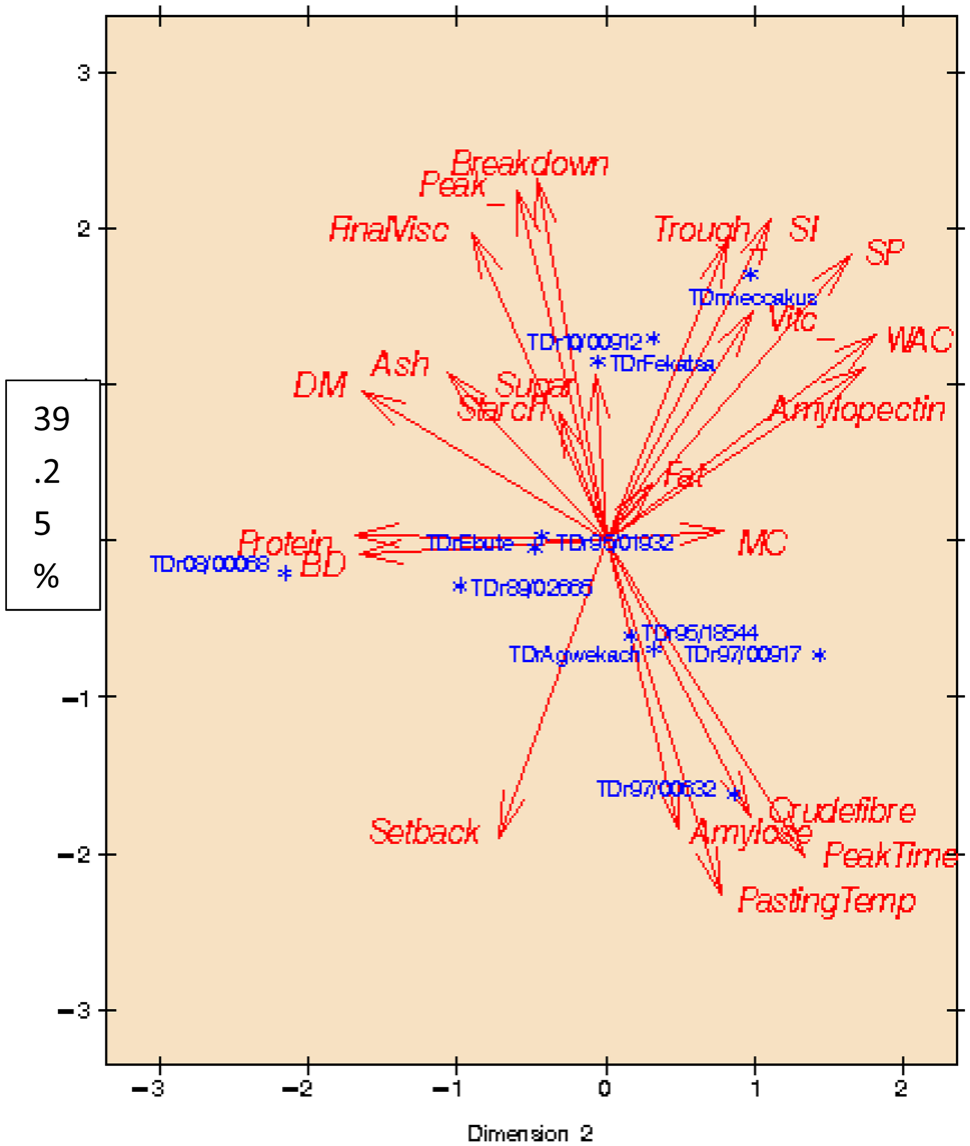
Principal Component Analysis of flour physicochemical and instant *fufu* product pasting attributes from *D. rotundata* control and improved varieties.

**Figure 3:**
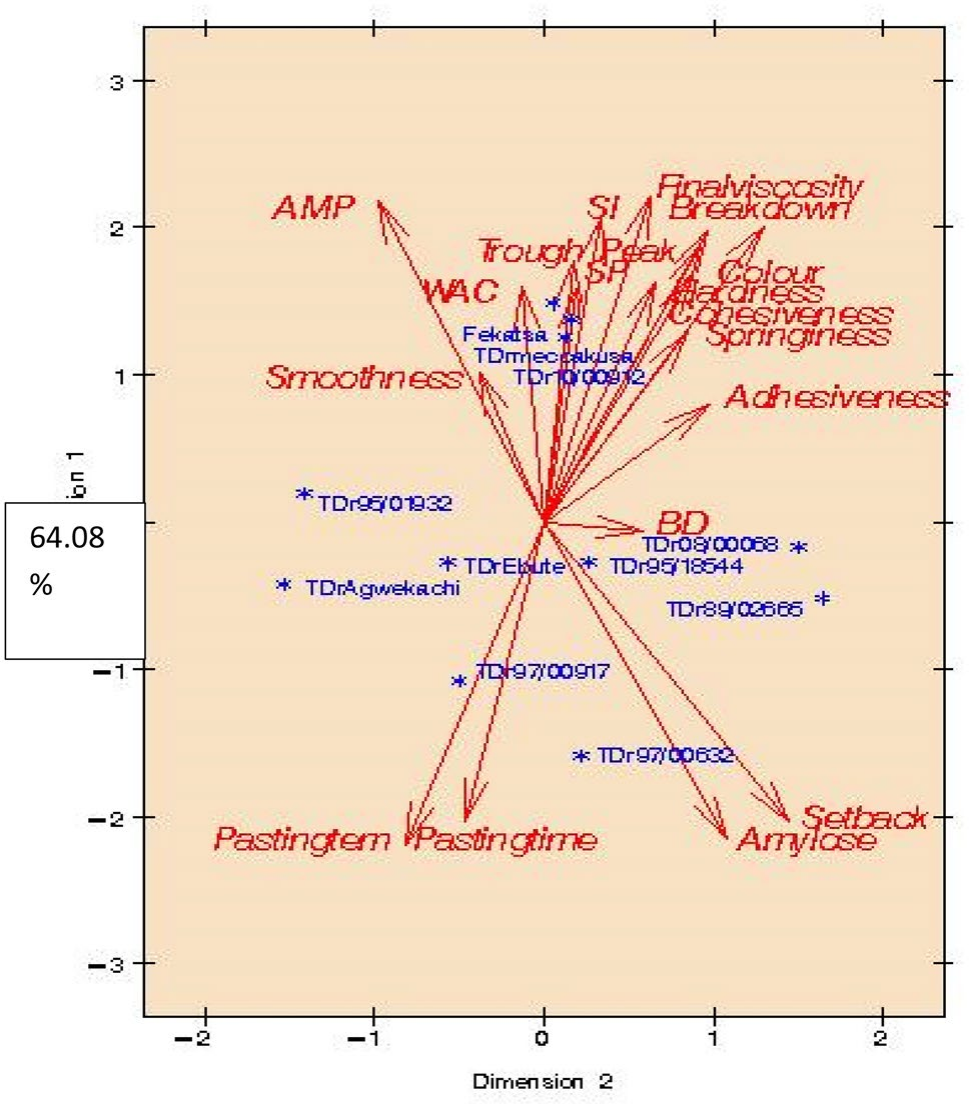
Principal Component Analysis of flour physicochemical with the *fufu* product pasting and sensory attributes from *D. rotundata* control and improved varieties.

Among the most frequently used multivariate data analysis methods, principal component analysis (PCA) aims to explain how much variance occurs within the analysed data matrix, by extracting the main orthogonal contributors (principal components). Considered a dimension-reduction technique, PCA helps to decrease a large set of variables to appear a small set, yet possessing most of the information found in the original set of variables^49^. The PCA of flour physicochemical loaded with *fufu* pasting, and sensory attributes obtained from control and improved varieties of *D. rotundata* are shown in Figs. 2 and 3. When the flour physicochemical is loaded with the *fufu* pasting only, as shown in Fig. 2, the PC axes 1 and 2 would explain 59.35% of the observed total variation. PC 1 accounted for 39.25% of the total variation, whereas the PC 2 accounted for 20.10%. At the positive end of PC 1, both TDr Meccakusa, TDr 10/00912 and *Fekatsa* appeared close to each other. Trough, solubility index, swelling power, water absorption capacity, amylopectin, fat and moisture content would contribute to their characteristic similarities. Quality characteristics of TDr Meccakusa and TDr 10/00912 appeared to compete with the control (*Fekatsa*).

However, when the flour's physicochemical was loaded with both *fufu* pasting and sensory attributes, as shown in Fig. 3, the dimensions 1 and 2 explained by 86.89% of the total variation were observed in the sample plot. The relationships that existed among the parameters observed in dimension 1 were explained by 64.08% of the variation, whereas those observed in dimension 2 were explained by 22.81% of the variation. The remaining 13.11% variations not accounted for in dimensions 1 and 2 might be due to some agronomical differences that existed among the yam varieties, and probably some experimental errors. Moreover, the comparable qualities observed in the loading of *Fekatsa* (control) appears closer to TDr Meccakusa and TDr 10/00912 explained by the observed relationships involving physicochemical (amylopectin, water absorption capacity, swelling power and solubility index), pasting characteristics (trough, peak, breakdown and final viscosities) and sensory(smoothness, color, hardness, cohesiveness, springiness and adhesiveness) parameters. Other improved yam varieties (TDr 08/00068, TDr Ebute, TDr 95/18544, TDr 89/02665, TDr Agwekachi, TDr 89/00917 and TDr 97/00632 showed comparable relationships among some pasting properties (pasting temperature/ time and setback viscosity) and their amylose property. Bulk density had more observed effect only on TDr 08/00068, which may not necessarily relate with the other physicochemical, pasting and sensory properties. The correlation coefficient results showing relationships of the studied parameters (Refer to Table 4) support the PCA outcome of the physicochemical, pasting, and sensory properties.

## Conclusion

The effects of yam varieties on flour physicochemical characteristics and resultant instant *fufu* pasting and sensory attributes were investigated. The study revealed that both TDr 10/00912 and TDr Meccakusa appear closely related to the control (*Fekatsa*). Fairly, the instant pounded yam prepared from TDr 10/00912, TDr Meccakusa, TDr 97/00632 and TDr 08/00068 compared well with those prepared from the control, *Fekatsa*. However, the PCA differentiations between the pasting (peak, trough, breakdown, and final viscosities), physicochemical (amylose, amylopectin contents, solubility index, swelling power, and water absorption capacity, bulk density), and sensory (color, smoothness, adhesiveness, cohesiveness, springiness, and hardness) parameters inclined towards the higher promise of TDr 10/00912 and TDr Meccakusa yam varieties. For emphasis, the quest to further understand how yam varieties would be effective on flour production, so as to help actualize a mealy, appropriately elastic, and smooth dough for the *fufu* product underpins the justification of this current work. Overall, the TDr 10/00912 and TDr Meccakusa are recommended and should be promoted, particularly to farmers, which would aim towards further implementation for commercialization of the instant pounded yam flour production for *fufu* that is more consistent, stable, and of acceptable quality.
